# Near-future rocket launches could slow ozone recovery

**DOI:** 10.1038/s41612-025-01098-6

**Published:** 2025-06-09

**Authors:** Laura E. Revell, Michele T. Bannister, Tyler F. M. Brown, Timofei Sukhodolov, Sandro Vattioni, John Dykema, David J. Frame, John Cater, Gabriel Chiodo, Eugene Rozanov

**Affiliations:** 1 School of Physical and Chemical Sciences–Te Kura Matū, University of Canterbury, Christchurch, New Zealand; 2 Physikalisch-Meteorologisches Observatorium Davos and World Radiation Center, Davos, Switzerland; 3 Institute for Atmospheric and Climate Science, ETH Zurich, Zurich, Switzerland; 4 John A. Paulson School of Engineering and Applied Sciences, Harvard University, Cambridge, MA USA; 5 Department of Mechanical Engineering, University of Canterbury, Christchurch, New Zealand; 6 Instituto de Geociencias(IGEO), CSIC-UCM, Madrid, Spain; 7 Ozone Layer and Upper Atmosphere Research Laboratory, Saint Petersburg State University, Saint Petersburg, Russia

**Keywords:** Atmospheric chemistry, Atmospheric dynamics, Business and industry

## Abstract

Rocket emissions thin the stratospheric ozone layer. To understand if significant ozone losses could occur as the launch industry grows, we examine two scenarios. Our ‘ambitious’ scenario (2040 launches/year) yields a −0.29% depletion in annual-mean, near-global total column ozone in 2030. Antarctic springtime ozone decreases by 3.9%. Our ‘conservative’ scenario (884 launches/year) yields −0.17% annual, near-global depletion; current licensing rates suggest this scenario may be exceeded before 2030. Ozone losses are driven by the chlorine produced from solid rocket motor propellant, and black carbon which is emitted from most propellants. The ozone layer is slowly healing from the effects of CFCs, yet global-mean ozone abundances are still 2% lower than measured prior to the onset of CFC-induced ozone depletion. Our results demonstrate that ongoing and frequent rocket launches could delay ozone recovery. Action is needed now to ensure that future growth of the launch industry and ozone protection are mutually sustainable.

## Introduction

**Fig. 1:**
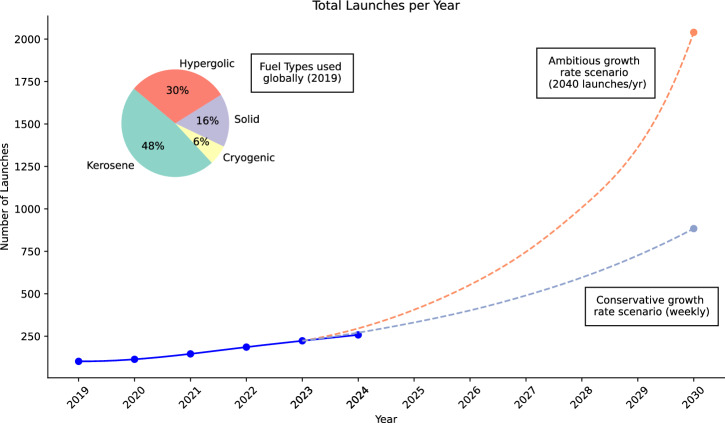
Rocket launches through 2024, with ambitious and conservative growth scenarios to 2030.

**Fig. 2:**
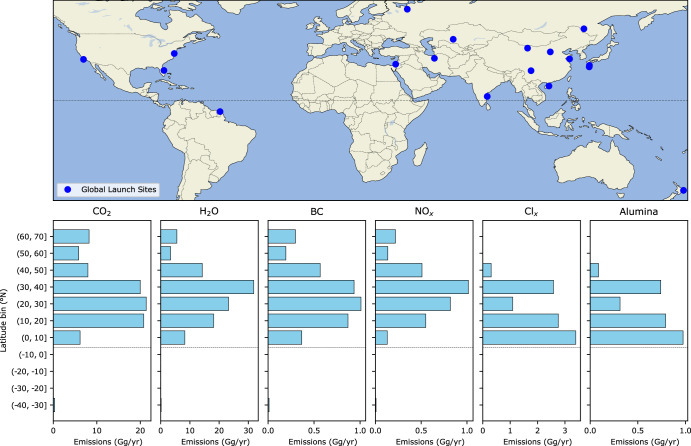
Map of launch sites and latitudinal distributions of annual emissions in the ambitious growth scenario. The dashed grey line represents the equator.

The past 5–10 years have seen a significant expansion of rocket launch activity. From 102 total launches worldwide in 2019, 2024 saw 258 orbital launches, with that number expected to be exceeded in 2025 (Fig. 1). The rocket launch cadence is determined by economic factors, including the rapid development of a near-Earth economy driven by both established providers and an ecosystem of enterprise often termed New Space^1^. One driver is the demand for launch of large-scale satellite constellations of thousands to tens of thousands of units into low-Earth orbit (LEO, ~250–600 km above Earth’s surface)^2^. The number of units in these constellations requires a high launch cadence for two reasons. First, as underway at present, for establishing each constellation. Second, for ongoing replenishment of the infrastructure, as units in LEO experience sufficient exospheric and thermospheric drag to rapidly (≤5–10 years) deorbit and re-enter the upper atmosphere, where it is intended by the operators that they ablate into small pieces. Worldwide, the geographic diversity of launch sites has also increased, though the great majority remain in the Northern Hemisphere (Fig. 2). Launch activity and locations have implications for their atmospheric impacts, discussed later on.

The combustion of rocket propellants to create thrust produces reactive gases and particulates, which are emitted into the atmosphere as a rocket traverses a flight profile up to space. Within this path, the stratosphere (~15–50 km above Earth’s surface) is where the ozone layer resides. Ozone protects the biosphere via absorption of solar UV-B radiation, plays a central role in maintaining the vertical temperature structure of the atmosphere, and has important implications for the surface circulation of both hemispheres^3–6^. Large ozone losses began to be observed in the late 20th century due to emissions of chlorofluorocarbons (CFCs) and other halocarbon gases. Thanks to the Montreal Protocol on Substances that Deplete the Ozone Layer and its later Amendments and adjustments, most halocarbons are now banned. The ozone layer is showing early signs of recovery^7^, with a return to 1980 levels projected for the next few decades, depending on latitude and future greenhouse gas emissions^8,9^.

Many of the gases and particulates produced by rockets are radiatively and/or chemically active with lifetimes of days to months, and can cause ozone destruction^10^. The principal emission species from propellants in common usage are carbon dioxide (CO_2_), water vapour (H_2_O), alumina (Al_2_O_3_) and black carbon particulates, reactive chlorine-containing species and nitrogen oxides (NO_x_=NO + NO_2_). Rocket exhaust products from the four most common propellant types (kerosene, cryogenic, hypergolic and solid rocket motor (SRM) propellant) can cause ozone depletion. Rocket launch-induced ozone depletion can occur either from launches^11–24^ or atmospheric re-entry of spacecraft^25,26^. However, launch cadences—and their expected growth—were substantially sparser and slower at the publication of previous works than they are today.

Two recent studies have examined the potential effects of contemporary rocket launch activities on the atmosphere. Maloney et al.^27^ modelled the impacts of rocket-emitted black carbon on the atmosphere and climate, and identified seasonally-dependent Northern Hemisphere ozone loss because of radiatively-driven warming of the stratosphere, which increases the chemical ozone loss rates. Ryan et al.^28^ modelled scenarios based on contemporary rocket launch activities and re-entry and identified global ozone losses that scale with increased launch cadence. Both studies acknowledged the uncertainties and scope for future work to quantify this emerging issue.

The 2022 WMO/UNEP Scientific Assessment of Ozone Depletion^29^ noted heightened concerns about the increased frequency of civilian rocket launches on 21st century stratospheric ozone—and large knowledge gaps. Here we use a coupled chemistry-climate model to study the impacts on ozone from rocket launches in the near-future (2030). We follow two near-future scenarios representing the global launch industry’s current aspirations, based on a precise benchmark of 2019s launch activity^30^ (see Methods).

## Results

**Fig. 3:**
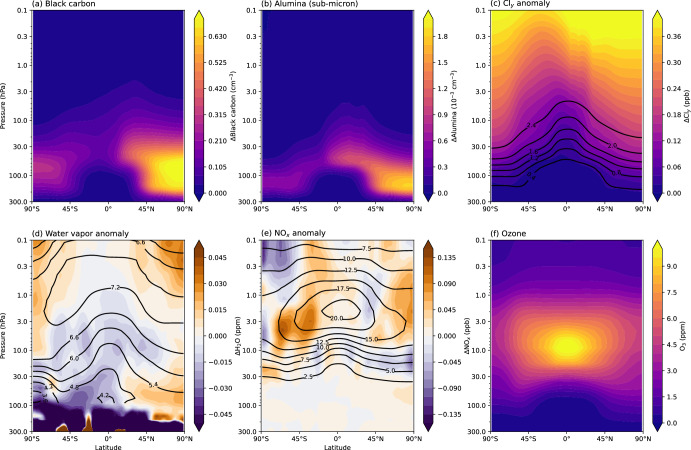
Distribution of rocket-distributed species in the stratosphere. **a**–**e** Concentration anomalies of annual, zonal-mean distribution of rocket-emitted species in the “ambitious growth” scenario. **a** Black carbon in the BC simulation; **b** Sub-micron fraction alumina in the ALL simulation; **c** Inorganic chlorine (Cl_y_ = HCl + ClONO_2_ + HOCl + ClO + 2 × Cl_2_O_2_ + Cl + 2 × Cl_2_ + BrCl) in the GAS simulation; **d** Water vapour in the GAS simulation; **e** Nitrogen oxides (NO_x_=NO+NO_2_) in the GAS simulation. Black contour lines in (**c**–**e**) show the background concentration of that species in the REF simulation (units of ppb for Cl_y_ and NO_x_ and ppm for water vapour). Black carbon and alumina are not present in the REF simulation, so their background concentration is not indicated in (**a**, **b**). **f** Ozone concentration in the REF simulation.

The simulated concentration anomalies of rocket-emitted species in 2030 are shown in Fig. 3. Black carbon and alumina are primarily emitted from launches that take place in the Northern Hemisphere (Fig. 2). Black carbon and alumina particles sediment to the lower stratosphere, reaching altitudes where gravitational sedimentation is compensated by the increased air density, and the particles can be transported to the Southern Hemisphere via the Brewer-Dobson circulation (Fig. 3a, b). Black carbon particles are of lower weight than alumina, and are therefore more spread out in the stratosphere. Inorganic chlorine (Cl_y_ = HCl + ClONO_2_ + HOCl + ClO + 2 × Cl_2_O_2_ + Cl + 2 × Cl_2_ + BrCl) increases by ~10% in the upper stratosphere. In contrast, changes in the concentrations of rocket-emitted water vapour and NO_x_ are small (less than 2% and 4%, respectively) and statistically insignificant compared with stratospheric background concentrations.

**Fig. 4:**
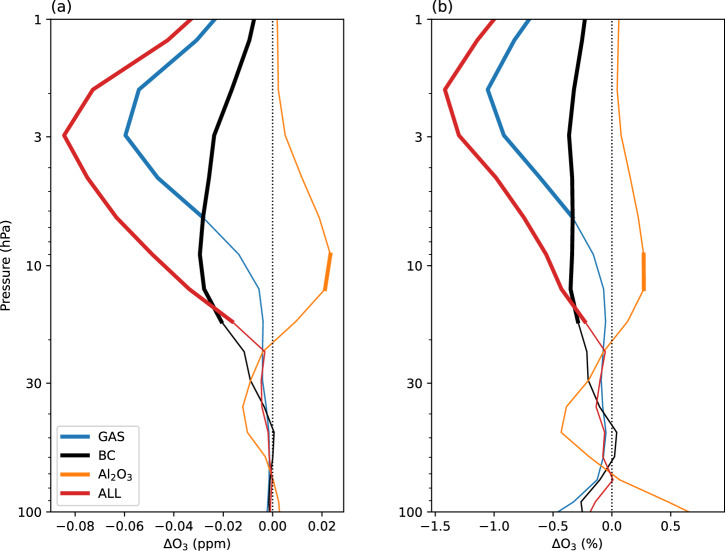
Influence of rocket-emitted gas-phase species, black carbon and alumina on ozone. Near-global (60°N-60°S) annual-mean ozone anomalies (i.e. relative to the REF simulation), shown as **a** an absolute difference; **b** a percent difference. Thick lines indicate where the difference is statistically significant (95% confidence interval).

**Fig. 5:**
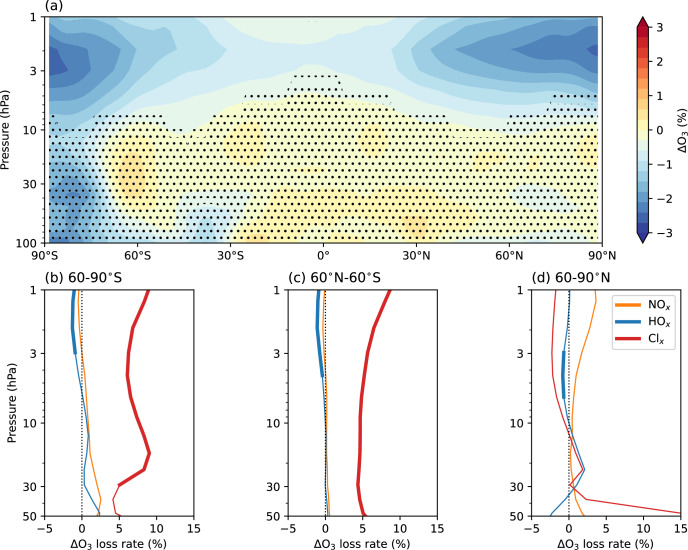
Changes to ozone and ozone-loss cycles from rocket-emitted gas-phase species. **a** Percent difference in annual-mean ozone concentration in the GAS simulation relative to the REF simulation. Stippling indicates where the difference is not statistically significant (95% confidence interval). Anomalies in annual-mean rates of ozone loss cycles in the GAS simulation relative to the REF simulation for **b** 60–90°S; **c** 60°N–60°S; **d** 60–90°N. Thick lines indicate where the difference is statistically significant (95% confidence interval).

**Fig. 6:**
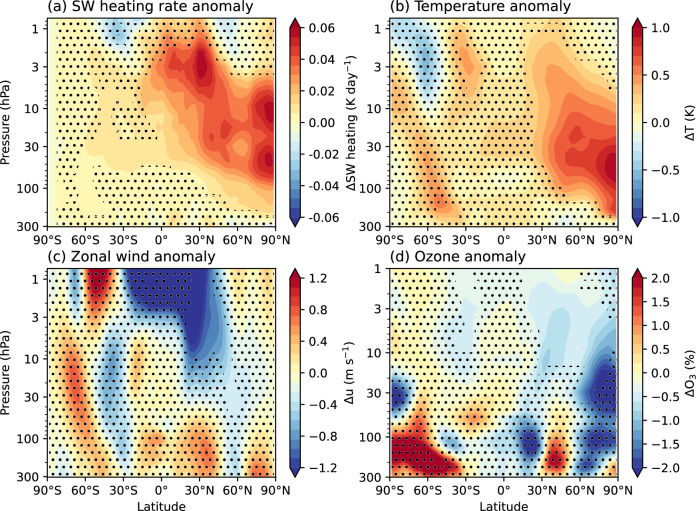
Influence of rocket-emitted black carbon on the stratosphere. Zonal-mean anomalies in the BC simulation (relative to the REF simulation) calculated for June-July-August (JJA) for: **a** the shortwave heating rate; **b** temperature; **c** zonal-mean wind speed; **d** ozone concentration. Stippling shows where the anomalies are not statistically significant (95% confidence interval).

Anomalies in near-global (60°N–60°S) ozone for the GAS, BC, Al_2_O_3_ and ALL simulations (see Methods) following the “ambitious growth” scenario are shown in Fig. 4. The largest impact is seen in the ALL simulation, where ozone decreases by up to 0.08 ppm, or 1.5%, in the upper stratosphere. Ozone loss is largely driven by reactive chlorine chemistry (Fig. 5) and the dynamical and radiative effects induced by black carbon (Fig. 6); these are discussed later on in this section. Alumina alone appears to have little impact on ozone in the quantities considered here, which is a useful point of comparison in the context of ablation of satellites re-entering the atmosphere (discussed later on). At most, a 0.27% increase in ozone is seen at 10 hPa in the Northern Hemisphere due to some acceleration of ozone transport by the shallow branch of the Brewer-Dobson circulation, following the alumina heating impacts on dynamics. Furthermore, the amounts of alumina considered here are relatively small. Vattioni et al.^31^ simulated a 10% ozone column reduction in stratospheric alumina geoengineering simulations with 5000 Gg of alumina injected annually. In contrast, we inject 18.32 Gg alumina in our Al_2_O_3_ simulation and 2.91 Gg in our ALL simulation (Table 1). We also used an “upper limit” parameterisation for the chlorine activation reaction R1 (see Methods). In reality, chlorine activation would be likely even smaller than in the simulations performed here. However, this still needs to be confirmed by laboratory experiments on the surface chemistry of alumina particles; their effects on polar stratospheric cloud formation and interaction with the sulfate aerosol layer are also very uncertain^31^.

**Table 1:** Annual stratospheric emission products from rocket launches in modelled scenarios

Simulation	Launch rate	NO_*x*_	Cl_*x*_	H_2_O	Black carbon	Alumina
	cadence	(Gg/yr)	(Gg/yr)	(Gg/yr)	(Gg/yr)	(Gg/yr)
Reference (REF)	n/a	0	0	0	0	0
Conservative growth						
All forcings	weekly	1.35	4.05	41.93	1.70	1.16^a^
Ambitious growth						
Gas phase products only (GAS)	72 h	3.38	10.13	104.82	0	0
Black carbon only (BC)	72 h	0	0	0	4.25	0
Alumina only (Al_2_O_3_)	72 h	0	0	0	0	18.32
All forcings (ALL)	72 h	3.38	10.13	104.82	4.25	2.91^a^

^a^Scaled to represent the assumed fraction of sub-micron alumina which persists for longer in the stratosphere (discussed in the “Methods” and ref. ^30^).

Compounding the above uncertainties, chemistry-climate models with horizontal resolutions of several hundred kilometres (see Methods) are not capable of spatially resolving rocket plumes with substantially higher local exhaust concentrations. Since heterogeneous chemistry is a strong non-linear function of the concentration of gaseous species such as HCl and H_2_O^31^, strong local ozone depletion via heterogeneous chemistry may occur^19^. Future work should investigate these aspects.

Chlorine-induced ozone loss causes the bulk of gas-phase ozone loss, demonstrated by the anomalies presented in Fig. 5 for the GAS simulation. The increased chlorine loading also leads to increased HCl concentrations (not shown), decreasing the availability of HO_*x*_ to participate in ozone depletion—seen in the small but statistically significant decrease in the rate of HO_x_-induced ozone loss (Fig. 5b–d). Because concentrations of rocket-emitted NO_*x*_ are insignificant compared with background concentrations, we see no appreciable change in the rate of NO_x_-induced ozone depletion (Fig. 5b–d).

Ozone decreases by up to 3% in the upper stratosphere (Fig. 5a), which is largely driven by a 5–10% increase in Cl_x_-induced ozone loss for the near-globe (60°N–60°S) and over Antarctica. Although all of the vehicles that produce chlorine (those using SRMs) are launched in the Northern Hemisphere (Fig. 2), the impacts propagate to the Southern Hemisphere due to the spread of Cl_y_ via the Brewer–Dobson circulation^32^. The increase in Cl_y_ in the Antarctic stratosphere (Fig. 3c) is due to transport via the deep branch of the Brewer–Dobson circulation. In addition, the meridional gradient in Cl_y_ in the Southern Hemisphere is steeper than in the Northern Hemisphere due to less wave dissipation and thus less efficient lateral mixing of tropical and polar air masses. Chlorine-induced ozone loss is enhanced over Antarctica but not the Arctic, due to the stronger polar vortex and the feedbacks between vortex temperature and isolation, polar stratospheric cloud formation, Cl_x_ activation and ozone depletion over Antarctica. While there is an increase in Cl_x_-induced ozone loss of ~15% in the Arctic lower stratosphere, it is not statistically significant due to the large variability in the Arctic.

Black carbon has significant impacts on ozone and climate at northern midlatitudes, where the majority of launches emitting it are made (Fig. 2). As seen in the BC simulation (Fig. 6), the largest impacts occur during Northern Hemisphere summer (June–July–August; JJA), when shortwave heating maximises. The stratosphere warms by up to 1 K during JJA. By warming the high latitudes, black carbon increases the meridional temperature gradient and strengthens the easterly winds in the subtropical upper stratosphere. The warming also leads to increases in the rate of NO_x_- and HO_x_-induced ozone destruction, and ozone loss of up to 2% (Fig. 6d).

**Fig. 7:**
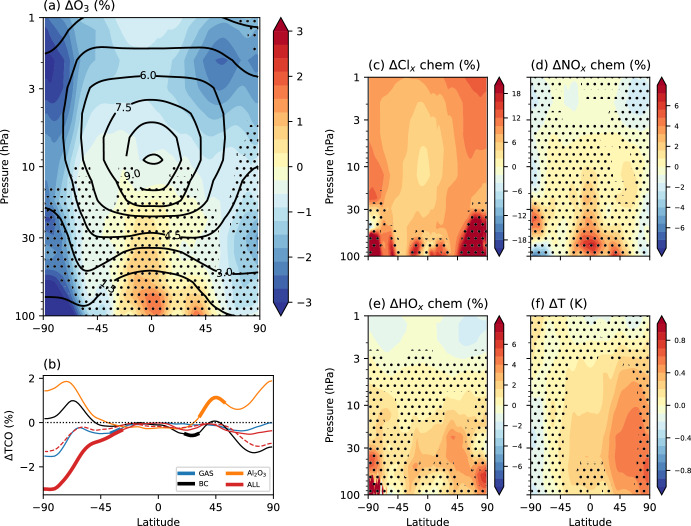
Overall effects of rocket emissions on the ozone layer. Combined effects of rocket emissions as an annual mean for the “ambitious growth” scenario (ALL relative to REF): **a** Ozone anomaly (as a percent). Black contour lines show the annual-mean ozone distribution in the REF simulation (units of ppm); **b** Total column ozone anomaly. Thick lines indicate where the anomaly is statistically significant (95% confidence interval). The solid red line shows the “ambitious growth” scenario with all forcings (ALL simulation) and the dashed red line shows the “conservative growth” scenario with all forcings; **c**–**e** Anomaly in the rate of the ozone-destroying Cl_x_, NO_x_ and HO_x_ cycles, respectively. **f** Temperature anomaly. In all panels except **b**, stippling indicates where the anomaly is not statistically significant (95% confidence interval).

**Fig. 8:**
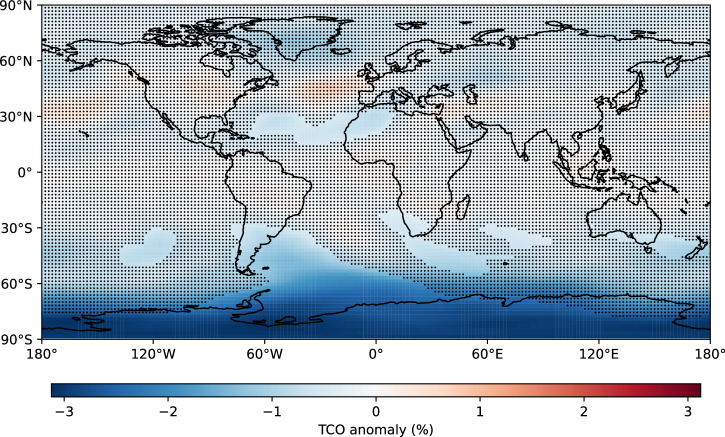
Annual-mean total column ozone anomaly (as a percent) in the “ambitious growth” scenario (ALL relative to REF). Stippling indicates where the anomaly is not statistically significant (95% confidence interval).

Statistically-significant ozone losses and stratospheric warming are seen in some regions of the atmosphere in both growth scenarios. In the ALL simulation representing ambitious growth with 2040 launches per year in 2030, upper stratospheric ozone decreases by ~3% (Fig. 7a). Statistically-significant impacts are seen across the Southern Hemisphere, where total column ozone decreases by up to 3% (Figs. 7b, 8). Seasonal Antarctic ozone losses are even larger (not shown): the increase in Cl_x_-induced ozone loss drives springtime ozone losses of 3.9% (95% confidence interval: −9.4% to +1.6%). Ozone losses of this order of magnitude are similar to the total column ozone loss (3–5%) that occurred at southern midlatitudes following the 2019–2020 Australian wildfires^33^. After episodic wildfires, ozone returns to background levels over the following months. In contrast, regularly scheduled rocket launches provide a recurring source of reactive gases and particulates to the stratosphere. In the ALL simulation, the effects on ozone are mostly due to Cl_y_ (from SRM-emitted HCl reacting) and black carbon (emitted from kerosene, SRM and hypergolic propellants).

Compared to the GAS simulation (Fig. 5a), the Southern Hemisphere lower-stratospheric ozone depletion is amplified in the ALL simulation (Fig. 7a) due to the additional polar vortex strengthening from the black carbon effects on tropical temperature that amplify the Southern Hemisphere meridional temperature gradient. Finally, an annual-mean stratospheric warming of ~0.6 K is seen in the Northern Hemisphere in the “ambitious growth” scenario, due to black carbon emission (Fig. 7f). A smaller warming of ~0.2 K is present in the “conservative growth” scenario (not shown).

The near-global (60°N–60°S) annual-mean total column ozone losses in both scenarios are consequential. Loss in the “ambitious growth” (ALL) simulation is 0.95 DU, or 0.29% (95% confidence interval: −0.54% to −0.04%). In the “conservative growth” simulation, ozone loss averages 0.56 DU, or 0.17% (95% confidence interval: −0.48% to +0.13%). The Montreal Protocol avoided widespread ozone damage by phasing out halocarbons. Nonetheless, the ozone layer is still recovering from the effects of these gases and is not projected to return to 1980 levels for several decades, depending on latitude and future greenhouse gas emissions^8^. Near-global (60°N–60°S) total column ozone measured in 2017–2020 was 2% smaller than the 1964–1980 average (i.e., prior to the onset of widespread halocarbon-induced ozone depletion) and increased at a rate of ~0.3% per decade over the 1996–2020 period^8,34^. Therefore, both scenarios presented here indicate that near-future rocket launches could jeopardise the benefits for ozone protection achieved through the Montreal Protocol.

## Discussion

Few studies are available in the era of New Space and contemporary rocket launches aside from two recent studies, which provide useful points of comparison^27,28^. Ryan et al.^28^ used the GEOS-Chem chemistry-transport model coupled to the RRTMG radiative transfer model to simulate a decade of 5.6% year-on-year growth on rates of 2019 rocket launches and spacecraft re-entry. They calculated a 0.01% decrease in global-mean total column ozone by the end of the decade relative to a simulation with no rocket launch or re-entry emissions. We see global-mean (90°N–90°S) total column ozone loss of 0.2 −0.4%. Our calculated ozone loss is an order of magnitude larger because our emissions are also approximately one order of magnitude larger (with the exception of NO_x_, as we omit re-entry NO_x_ from our simulations). For example, our “ambitious growth” scenario assumes emission of 10.13 Gg Cl_*x*_, 104.82 Gg H_2_O, 4.25 Gg black carbon, 3.38 Gg NO_*x*_ and 2.91 Gg sub-micron alumina. In contrast, by the end of their 5.6%/year growth scenario, Ryan et al. assumes emission of 1.4 Gg Cl_*x*_, 16.7 Gg H_2_O, 0.8 Gg black carbon, 3.4 Gg NO_x_ and 2.5 Gg sub-micron alumina.

Maloney et al. used the CESM2-WACCM6 Earth System Model to investigate the effects on ozone and climate of varying quantities of rocket-emitted black carbon^27^. With a 10 Gg/year injection of black carbon at 30°N they projected ozone loss of up to 16 DU in the Northern Hemisphere. Our results are consistent with their findings when accounting for differences in approach—in their 10 Gg/year black carbon scenario, JJA shortwave heating anomalies simulated in the Northern Hemisphere were up to 10 K/season and the stratosphere warmed by up to 1.4 K. JJA easterly winds in the upper stratosphere strengthened by up to 5 m s^−1^. In our “ambitious growth” and BC simulations, we emitted 4.3 Gg year^−1^ of black carbon from all 17 sites, of which 16 are located in the Northern Hemisphere. Black carbon exerted the largest effects during the Northern Hemisphere summer when incoming solar radiation maximises. In JJA we found shortwave heating anomalies of up to 0.06 K day^−1^ (corresponding to 4.5 K/season, for comparison with ref. ^27^), warming up to 1 K, and strengthening of the upper stratospheric easterly winds of up to 1.2 m s^−1^ (Fig. 6). Similar to Maloney et al., black carbon-induced ozone loss in our simulations originates from stratospheric heating and dynamics, rather than heterogeneous chemical reactions involving black carbon.

While there are many uncertainties in rocket emission impacts on the stratosphere, the comparisons between these three studies on New Space launches performed with modern climate models illustrate some notable consistencies:

1. Chlorine and black carbon emitted from rocket propellants cause ozone depletion
2. Black carbon also causes atmospheric warming
3. Increasing the launch emissions inputs creates bigger impacts
4. The impacts are not uniformly distributed: they depend on where launches take place, how often, what vehicles are in use at that site and what propellant types are used.
5. Launches from a single site can have global impacts.

The emerging results of scientific analysis suggests that at least one current industry practice should change to ensure the world’s ozone recovery is sustained. First, the use of rocket propellants that lead to chlorine emissions in the stratosphere needs close attention by scientists and regulators. This is produced by SRMs (e.g. boosters for hydrolox rockets) that co-combust aluminium and HCl; the alumina particles act as reaction surfaces for chlorine to accelerate ozone loss. A driving factor in the ozone loss shown here is the proportion of SRMs in world use. Avoiding SRM growth at the levels shown in our scenarios would support ozone protection.

Second, the use of large quantities of rocket propellants that lead to black carbon emissions in the stratosphere needs careful consideration and further study. Black carbon causes ozone depletion and atmospheric warming, both of which can have important implications forstratospheric dynamics, stratosphere-troposphere coupling, and surface regional climate^5,35,36^. Propulsion engineers should test for black carbon and try to minimise its emission in the stratosphere to reduce its effects on ozone. In Brown et al.^10^, we identified five actions that the aerospace industry could take, including quantifying launch vehicle emissions at both the design and testing stages, and the promotion and normalisation of emissions data availability, which aids collaboration with the stratospheric modelling community. In the absence of clear knowledge about the role of massively increasing black carbon inputs, for instance if new heavy-lift “methalox” LNG vehicles launch weekly, these actions will be key to defining an ozone-safe operating envelope for such vehicles.

As noted earlier, future launch rates are uncertain, but economic factors are driving their acceleration. Greater impacts on ozone recovery come from more launches, or fewer, more highly emitting launches. We do not suggest a cessation of launches, but consideration of stratospheric effects in the operation of launches is key^37^. A degree of global coordination in propellant type usage could help. Industry could potentially shift the mix of launches from the 2019 ratio we apply here(slight shifts are present in 2020–22 ref. ^38^), while future launch vehicles could introduce different propellant types. However, we emphasise that propellant types in current, active use have clear projected effects in delaying near-future ozone recovery.

Regulators and other policymakers also need to pay close attention to the stratospheric impacts of rocket launches, if continued ozone recovery is to be assured. Because the ozone-depleting products produced by rocket launches are short-lived in the stratosphere—either because they are reactive species or because they soon fall to lower altitudes—they are in general non-uniformly-mixed flow pollutants. The degree to which launch pollutants can be approximated for policy purposes as regionally well-mixed is something that requires more research. There may be some regions in which pollutants are uniformly-mixed, over some relevant timescales (Fig. 3). In these regions it would make sense to regulate across their launch sites. In regions where impacts do not pool in this way, it may make sense to regulate on a source-by-source basis. In essence, though, the standard tools of environmental policy should be brought to bear on the problem to protect the ozone layer and its recovery. Sites—either globally, regionally, or separately—could apply a cap and trade approach to pollutants, or to launches, depending on the fungibility of the pollutants.

In theory a price-based system could be applied instead of a quantity-based system. However, Weitzman^39^ makes the argument that complex manufacturing processes and other “situations demanding a high degree of coordination” usually imply a preference for quantity-based instruments (permits) rather than price-based instruments(taxes or fees). The argument is that complex and specialised production processes create kinks in the benefit function associated with emissions reductions. “The resulting strong curvature in benefits around the planned consumption level tends to create a very high comparative advantage for quantity instruments.” In the present case, given the complexity and cost of rocket production, it is not clear that a flat fee associated with emissions would yield the desired emissions reductions. Many manufacturers may either pass on the cost or compete to make cost-savings elsewhere, doing little to reduce emissions. On the other hand, a quantity-based approach fixes the quantity emitted, and allows costs to settle in response. Some have disputed the generality of Weitzman’s claim above^40^, but in the case of ozone destruction from rocket launches, Weitzman’s argument appears persuasive.

We acknowledge large uncertainties associated with the launch cadences simulated here; our scenarios were constructed against a backdrop of rapid and evolving development. Our scenarios were designed to demonstrate outcomes from potential upper and lower bounds to the available parameter space. We select a four-fold and ten-fold increase in signal to quantify more severe effects of the known inputs. For perspective, 70 launches took place in 2010. From 97 launches providing stratospheric emissions inputs in 2019, there were 104, 135, 178 and 211 successful launches in each successive year through 2023^41^. In our lower bound, the conservative scenario, the 884 launches are a four-fold increase on 2023’s launches. In 2024, several launch operators already achieved cadences with intervals of repeat launches within a week. The conservative growth scenario thus serves as a reasonable expectation, and one which may be on track for being exceeded; e.g in U.S. launches, a threefold increase in launches over those in U.S. Financial Year 2023 is expected as soon as 2028^42^. For instance, if a 2030 global constellation steady-state reaches 100,000 active satellites, with 22,000 reaching end of life each year, then 733 launches annually of 30 larger satellites would be required for maintenance; with a constellation growth of 30% still underway, one could expect 953 launches/year.

The rate of rocket launches is largely limited by logistics and resources, i.e. an increase in launch sites or injection of significant financial capital. Given this unbounded projection space, we have opted to look towards existing government regulation to help guide realism in a growth scenario. New Zealand is one of the few nations which has published an upper limit on launches, currently regulated to one launch every 72 h. Using this existing regulation as a guide, our ‘ambitious scenario’ projections conveniently approximate a 10× increase from 2023 rates—providing both an order-of-magnitude input signal increase and extrapolation based on real current legislation. While a 72-h cadence happening across all pads at a given launch complex is presently challenging for operators, it is certainly within the envelope of industry discussions and aspirations by 2030—particularly for major providers^43^, whose outputs dominate emission products. One could also consider a situation where more or larger launch complexes exist in 2030 at a similar geographic distribution to the 17 we use here, with our 2040 launches of 2030 spread accordingly: launch providers often expand or refurbish existing facilities over greenfield sites, and several active sites in our simulations here fall within the same model cell. Already in 2023 there were 25 launch complexes in use worldwide^41^. Variations on our choices are entirely plausible and will be explored in future work.

Our results demonstrate that rocket launch emissions could have ongoing and significant effects on the ozone layer. However, we have only examined effects from launches and not re-entry material, which is important to consider in future work. The two re-entry species of primary concern are NO_x_ and alumina.

As well as being emitted propellants in contemporary use, NO_x_ is produced in the shockwave of objects re-entering the atmosphere^25^. It can be estimated from the re-entering object’s velocity, trajectory, surface area and mass^44^, but only mass is easily available^28^. We did not include re-entry NO_x_ due to data unavailability at the time of modelling^30^. Estimates of NO_*x*_ created from spacecraft and space debris re-entry heating are substantially larger compared with launch emissions^17^. In a study of 2019 rocket launches and re-entry, it was conservatively identified that ~95% of NO_x_ emissions were from re-entry, with the remainder from launches^28^, though this may be sensitive to the value of the re-entry emission index^38^.

We note that not all vehicles in our scenarios are designed for reusability: in our “ambitious growth” scenario, only 4.4% of launches are using vehicles designed for re-entry. However, the arc of industry practise is bending toward partial re-use of launch vehicles, where initial stages retain a small fraction of fuel and burn it in controlled re-entries from suborbital space to land softly. Drivers for this practice include cost-benefit analysis, life-cycle material and carbon conservation, and improved operational efficiency, so routine re-use is expected to become more significant in future vehicle designs^45^. Demise of LEO satellite constellations will also increasingly contribute NO_*x*_^38^, but the levels are uncertain.

Alumina from the eventual ablation in the upper atmosphere of the launches’ payloads, satellite constellations, has led to discussions of potential ozone impacts^46–49^. Alumina is not naturally present in the stratosphere in appreciable quantities, with some 55 kg ablated from dust particles mainly from Halley-type comets, a tiny percentage of the 28,000 ± 16,000 kg total daily meteor mass^50,51^. As LEO constellations are designed with a philosophy of infrastructure maintenance by ongoing replenishment and constant demise, their alumina inputs are projected to increase through 2030. The deorbit rates (input flux) remain uncertain, due to the wide scope of constellation operator behaviour, space weather, and anthropogenically-induced thermosphere contraction e.g. ref. ^52^. At minimum, near-Earth drag rates mean the current 9692 satellites in <600 km orbits(as of 2025-03-04; ref. ^41^) will largely re-enter within 5–10 years. Under similar assumptions to ref. ^53^ and placing it all in submicron alumina, this could perhaps reach 0.2 Gg/yr. The scaled growth scenarios of^53^ reach 0.8–2.5 Gg/yr; including reentering boosters may reach 5 Gg/yr^38^. We do not model re-entry alumina here. However, we do not see appreciable ozone loss from launch inputs of ~18 Gg/yr. A recent satellite re-entry study focussing on radiative effects suggests that deorbit rates of 10 Gg/yr (corresponding to a 60,000-LEO satellite population by 2040) could lead to an accumulated burden of 20–40 Gg of Al_2_O_3_ aerosol at 10–30 km over extratropical latitudes^49^. Radiative heating of the mesosphere and stratosphere occurs as a result, accompanied by small perturbations to winds and stratospheric ozone. As ref. ^49^ acknowledge, future modelling requires simulation of dynamical, chemical and radiative impacts to understand the full magnitude of the coupled effects.

The projected ozone losses reported here demonstrate that, consistent with prior work, increasing launch emissions will lead to near-future increasing ozone destruction, at a time when ozone should be recovering from the effects of CFCs and other ozone-depleting gases banned under the Montreal Protocol. The use of propellants in SRMs producing chlorine emissions needs immediate careful assessment by the global community. Fuel types leading to black carbon emission need ongoing quantification and minimisation.

Launches are created locally, yet lead to global impact. Creativity and aspiration across nations drove humanity’s desire to go to space. Creating a future supporting both industry growth and protection of a biosphere-critical part of the planet will be worthy of these dreams.

## Methods

Understanding the various perturbations that rocket launch emissions could have on the ozone layer requires a model that represents the coupled feedbacks between chemistry and climate. Chemistry-climate models (CCMs) are ideally suited to this purpose. CCMs have a dynamical core that is interactively coupled to a detailed chemistry scheme^54^. Chemical processes change the chemical composition of the atmosphere, which affects radiative heating and cooling, and consequently dynamics and radiation balance, while dynamical processes affect chemistry via temperature changes and transport. Here we used a CCM which represents the interactive coupling between atmospheric chemistry, dynamics and radiative processes, and additionally has a sophisticated aerosol scheme and interactive ocean.

### Model description

Simulations were performed with the SOCOLv4 (Solar-Climate Ozone Links version 4) atmosphere-ocean-aerosol-chemistry-climate model^55^. SOCOLv4 is based on the interactive coupling of the Max Planck Institute Earth System Model version 1.2 (MPI-ESM1.2)^56^, the MEZON chemistry model^57^ and a redesigned version of the sectional sulfate aerosol microphysical model AER, originally published by Weisenstein et al. ^58^. SOCOLv4 uses T63 horizontal resolution, corresponding to an approximate grid spacing of 1.9° × 1.9°. The atmosphere contains 47 vertical levels from the surface to 0.01 hPa (~80 km) using a hybrid sigma-pressure coordinate system, with a dynamical time step of 7.5 min and a chemical time step of 2 h. The model includes tracers for 99 chemical gas species as well as sulfuric acid aerosols and accounts for the most relevant reactions in atmospheric chemistry. The transport of chemical trace species is calculated with a semi-Lagrangian mass conservation scheme which is part of the ECHAM6 module^59^. Overall, the model produces accurate simulations of stratospheric ozone compared with observations^55^. SOCOL4 and its predecessor models are established tools for assessing the impacts of novel forcings on the ozone layer^36,60–62^, and have informed past WMO/UNEP Scientific Assessments of Ozone Depletion^63^.

To help understand the influence of gas-phase rocket emissions on stratospheric ozone, the reaction rates of the ozone-depleting NO_x_(NO_x_=NO+NO_2_), HO_x_(HO_x _= H+OH+HO_2_) and Cl_x_(Cl_x _= Cl + ClO) cycles were saved in every model grid cell^64–66^. This approach allows key reactions for the ozone budget to be analysed as a function of latitude, longitude, pressure and time.

SOCOLv4 was recently updated with a solid particle microphysics scheme to allow for simulation of climate engineering via stratospheric aerosol injection of solid particles^67^. We make use of this scheme for the black carbon and alumina particle emissions. Black carbon particles from rocket launches using kerosene, solid fuels and hypergolic propellants are represented as monodisperse spheres of radius 120 nm, and density of 1.8 g cm^−3^ ^68^. Black carbon particles emitted from combustion processes typically have sizes of tens to hundreds of nm^69,70^. Research using an earlier version of SOCOL simulated black carbon particles with radii of 50 nm and 100 nm and found no significant difference in simulated temperature and ozone distributions attributable to particle size^71^. Similarly, simulations with black carbon particles of radii 80 nm and 150 nm produced similar results to one another^35^. Significantly more radiative heating of the stratosphere and ozone losses occurred when the particle radius decreased to 30 nm^35^. The black carbon results presented here thus represent a lower bound—that is, we may see larger effects on temperature and ozone if the particles are significantly smaller than the 120 nm radius assumed here. Future work should focus on accurately quantifying the particle size distribution from rocket exhaust.

Because SOCOLv4 treats black carbon particles as chemically inert, all effects seen are due to heating, following the refractive indices of ref. ^72^ and Mie-scattering theory calculations^67^. In reality, black carbon can contribute to heterogeneous chemistry via the acquisition of sulfate coating as it ages, thereby contributing to the surface area density of sulfate aerosol^73^. Nonetheless, previous research indicates that the dominant effect on ozone of rocket-emitted black carbon is via stratospheric heating and dynamical impacts, rather than chemical impacts^27^.

Alumina (Al_2_O_3_) particles from SRMs are assumed to be spheres with radius 215 nm, which is well within the range of estimates from in-plume sampling^23,74,75^. Alumina particles have a density of 3.95 g cm^−3^, which can further coagulate and form higher order agglomerates (1, 2, 4, 8 and 16-mers)^67^. Both alumina and black carbon particles are subject to gravitational sedimentation and removal by SOCOLv4’s wet and dry deposition schemes. Alumina particles in SOCOLv4 are radiatively active, using the refractive indices of ref. ^76^. We also assume that alumina provides the surface for the heterogeneous reaction:

$${{\rm{ClONO}}}_{2}({\rm{g}})+{\rm{HCl}}({\rm{g}})\mathop{\longrightarrow }\limits^{{\rm{surf}}}{{\rm{Cl}}}_{2}({\rm{g}})+{{\rm{HNO}}}_{3}({\rm{g}})$$

which contributes to activation of stratospheric chlorine to a reactive, ozone-depleting form. The reaction probability *γ*_ClONO2_ was experimentally measured at stratospheric temperatures^77^. We use a Langmuir-Hinshelwood description of adsorption and reaction to extrapolate the measured uptake coefficients to stratospheric HCl partial pressures^31^. For extrapolation to stratospheric HCl partial pressures we applied the most conservative “upper limit” Langmuir-Hinshelwood fit assuming no dissociation at the particle surface and no co-adsorption of HNO_3_, which is the “high, non-dissociative *γ*_ClONO2_” scenario in ref. ^31^. This is a valid assumption since the measurements were originally performed assuming elevated HCl partial pressures as they would occur in SRM exhaust plumes^78^.

### Simulations

We performed 25-year time-slice simulations for the year 2030. Time-slice simulations use annually repeating boundary conditions which allow natural variability to be suppressed in the derivation of the rocket signals. Boundary conditions correspond to those required for the refD2 scenario designed for phase 2 of the Chemistry-Climate Model Initiative^79^. That is, greenhouse gas concentrations follow the 6th Coupled Model Intercomparison Project (CMIP6) SSP2-4.5 “reference future” scenario^80^ and ozone-depleting substances follow the WMO 2018 scenario, which assumes a phase-out of anthropogenic halocarbon gases following the Montreal Protocol^81^.

Anthropogenic and natural surface emissions of NO_x_ (including aircraft NO_x_), CO, and other organic compounds were defined from the CMIP6 SSP2-4.5 scenario^55^. NO_x_ production from energetic particles is calculated in SOCOL4 using daily ionisation rates from CMIP6, and daily geomagnetic Ap indices are used to calculate the influx of thermospheric NO_x_ through the top model layer (80 km).

Simulations were branched in 2030 from an already-performed refD2 simulation^82^. For the reference simulation and the experiments with rocket emissions (Table 1), the model was run for 35 years (keeping boundary conditions for the year 2030). The first 10 years were discarded as spin-up, as is typically recommended for chemistry-climate models^83^.

Rocket launch emissions were prescribed using an inventory that includes all vehicles that were active worldwide as of 2019^30^. These vehicles use four principal rocket propellants: kerosene (also called RP-1), hypergolic, cryogenic and SRM fuel. All these propellants create emissions that are relevant to ozone (Brown et al.^10^; see also that work’s Table 1 and Box 1 for a summary). In brief, kerosene produces carbon dioxide (CO_2_), water vapour (H_2_O), nitrogen oxides (NO_x_ = NO + NO_2_) and black carbon emissions. Hypergolic fuel produces CO_2_, H_2_O, NO_x_ and black carbon, and cryogenic fuel produces H_2_O, NO_x_ and hydrogen gas. SRM fuel produces CO_2_, H_2_O, NO_x_, black carbon, alumina (Al_2_O_3_) particulates and hydrogen chloride (HCl) emissions. The latter is converted rapidly to reactive chlorine (Cl_x_=Cl + ClO) in the stratosphere via heterogeneous reactions on the surface of particles, including polar stratospheric clouds, sulfate aerosols, and alumina particles.

Emissions for one of our scenarios as a function of geographic location are shown in Fig. 2. Six SOCOLv4 simulations were performed to explore the sensitivity of stratospheric ozone to the variety of rocket launch emission products. The total emissions products included in each are specified in Table 1. We ran a reference simulation with no launches included that we term REF; a sensitivity simulation of all forcings in combination under a conservative growth of rocket launches by 2030; and four separate sensitivity simulations with an ambitious growth of launches by 2030. In the ‘ambitious’ set of simulations, one simulation included only the gas-phase emission products from rocket launches (GAS: NO_x_, Cl_x_ and H_2_O), another included only chemically inert black carbon (BC), and another only alumina (Al_2_O_3_). The final “ambitious” simulation included all forcings in combination (ALL). Year-to-year variability in ozone, Cl_x_, NO_x_, HO_x_ and temperature throughout the 25-year ALL simulation are shown in Supplementary Fig. 1. For both growth scenarios’ ALL simulations, the total alumina burden was scaled (i.e. divided by 6.3) to represent only sub-micron-fraction alumina. Rocket emissions may also involve larger alumina particulates^30^, with a poorly quantified size distribution that we estimate via an emission index. The Al_2_O_3_ simulation did not apply such scaling and was designed to quantify the sensitivity of ozone to particulate alumina. CO_2_ emissions from rocket launches are negligible compared with other anthropogenic sources^22^, and are not further investigated here. Re-entry NO_x_ emissions are also not included, due to data unavailability for many currently used vehicles.

We set the worldwide ratios of fuels in use across launch sites as constant, using a customised version of the rocket launch emissions in the inventory of Brown et al.^30^. The exact list of vehicles is given in Table S1 in Supplementary Information. Emissions are gridded as a function of time (monthly mean), pressure, latitude and longitude. Brown et al.^30^ provide a netCDF file of the global rocket launches that placed emissions into the stratosphere in 2019, for use in launch scenario modelling. We use a modification of this file to create our two launch rate scenarios. Following the approach of ref. ^30^, the rate of emission per kilometre was calculated from the emission inventory and interpolated to the SOCOLv4 pressure grid. Emissions profiles are provided to the model over its entire altitude range (0–80 km). Note that the emissions we provide between 50 and 80 km follow an exponential decay, due to data availability constraints upward of 50 km^30^.

We explored two rates of launch frequency growth by 2030: termed ambitious and conservative (Fig. 1). The “ambitious” simulations used a frequency equivalent to a 72-h launch cadence for vehicles in use as of 2019 at each of 17 active launch sites, equating to 2040 launches per year (Fig. 2). The “conservative” scenario is equivalent to a weekly launch cadence at these sites (884 launches/year). Note that the input to the model (Table S1 in the Supplementary Information) is a monthly mean of emissions at each geographic coordinate: the simulation is agnostic to the fine temporal details of a given launch complex’s exact cadence of operation or number of pads in 2030, just that the emissions take place in a given month to the same proportions of fuel types as those in use in 2019. The same approach is used for treating aircraft emissions in chemistry-climate models^84^.

## Supplementary information

Supplementary Information

## Data Availability

SOCOL4 simulation data are available at^85^: https://zenodo.org/records/14183405.

## Code availability

SOCOL4 model code is available at^86^: https://zenodo.org/records/11073387.
