# Supplementary material for: Near-future rocket launches could slow ozone recovery
Source: NPJ Clim Atmos Sci. 2025 Jun 9;8(1):212. doi: 10.1038/s41612-025-01098-6 (PMC12148926; doi:10.1038/s41612-025-01098-6)
Supplement: Supplementary file 1 — Supplementary Information [file 41612_2025_1098_MOESM1_ESM.pdf]

Supporting Information for: Near-future rocket launches could slow ozone recovery

Laura E. Revell[1,*], Michele T. Bannister[1,*], Tyler F. M. Brown[1], Timofei Sukhodolov[2], Sandro Vattioni[3,4], John Dykema[4], David J. Frame[1], John Cater[5], Gabriel Chiodo[6], and Eugene Rozanov[2,7]

[1]School of Physical and Chemical Sciences–Te Kura Matū, University of Canterbury, Christchurch, Aotearoa New Zealand

[2]Physikalisch-Meteorologisches Observatorium Davos and World Radiation Center, Davos, Switzerland

[3]Institute for Atmospheric and Climate Science, ETH Zurich, Zurich, Switzerland

[4]John A. Paulson School of Engineering and Applied Sciences, Harvard University, Cambridge, MA, USA

[5]Department of Mechanical Engineering, University of Canterbury, Christchurch, Aotearoa New Zealand

[6]Instituto de Geociencias (IGEO), CSIC-UCM, Madrid, Spain

[7]Ozone Layer and Upper Atmosphere Research Laboratory, Saint Petersburg State University, Russian Federation

[*]*Corresponding authors, contact: laura.revell@canterbury.ac.nz and michele.bannister@canterbury.ac.nz*

Table 1: Inventory of launches in the “ambitious growth” scenario

Vehicle	Total Launches	Launch Site(s)	Fuel Type (main)	Fuel Type (booster)
Angara A5	40	Plesetsk	Kerosene	Kerosene
Rokot	40	Plesetsk, Baikonur	Hypergolic	-
Soyuz-2.1v	100	Plesetsk, Baikonur, Vostochny	Kerosene	-
Soyuz-2.1a	70	Plesetsk, Baikonur, Vostochny	Kerosene	Kerosene
Soyuz-2.1b	70	Plesetsk, Baikonur, Vostochny	Kerosene	Kerosene
Proton-M	20	Baikonur	Hypergolic	-
Proton-M+	20	Baikonur	Hypergolic	-
Atlas V 551	20	Cape Canaveral, Vandenberg	Kerosene	Solid
Atlas V N22	20	Cape Canaveral, Vandenberg	Kerosene	Solid
Delta IV M+ (4,2)	10	Cape Canaveral, Vandenberg	Cryogenic	Solid
Delta IV M+ (5,4)	30	Cape Canaveral, Vandenberg	Cryogenic	Solid
Falcon 9	60	Cape Canaveral, Vandenberg	Kerosene	-
Falcon Heavy	30	Cape Canaveral	Kerosene	Kerosene
Delta IV Heavy	50	Cape Canaveral, Vandenberg	Cryogenic	Cryogenic
Minotaur V	10	Vandenberg, Wallops	Solid	Solid
Ariane 5 ECA	50	Kourou	Cryogenic	Solid
Soyuz ST-B	10	Kourou	Kerosene	Kerosene
Vega	50	Kourou	Solid	-
Long March 2C	50	Jiuquan, Taiyuan, Xichang	Hypergolic	-
Long March 2D	50	Jiuquan, Taiyuan, Xichang	Hypergolic	-
Long March 2F	50	Jiuquan	Hypergolic	Hypergolic
Long March 3A	20	Xichang	Hypergolic	-
Long March 3B/E	20	Xichang	Hypergolic	Hypergolic
Long March 3C/E	20	Xichang	Hypergolic	Hypergolic
Kuaizhou 11	60	Jiuquan, Taiyuan, Yellow Sea	Solid	-
Long March 4B	30	Jiuquan, Taiyuan	Hypergolic	-
Long March 4C	30	Jiuquan, Taiyuan, Xichang	Hypergolic	-
Long March 11	15	Jiuquan, Xichang	Solid	Solid
H-IIA 204	40	Tanegashima	Cryogenic	Solid
H-IIB	80	Tanegashima	Cryogenic	Solid
Long March 6	15	Taiyuan	Kerosene	-
GSLV Mk II	30	Satish Dawan	Solid	Hypergolic
GSLV Mk III	30	Satish Dawan	Hypergolic	Solid
PSLV-CA	10	Satish Dawan	Solid	-
PSLV-DL	10	Satish Dawan	Solid	Solid
PSLV-QL	20	Satish Dawan	Solid	Solid
PSLV-XL	20	Satish Dawan	Solid	Solid
Antares 230	60	Wallops	Kerosene	-
Antares 230+	60	Wallops	Kerosene	-
Epsilon	120	Uchinoura	Solid	-
Shavit	120	Palmachin	Solid	-
Qased	40	Semnan	Hypergolic	-
Safir	80	Semnan	Hypergolic	-
Electron	120	Māhia	Kerosene	-
Long March 5	20	Wenchang	Cryogenic	Kerosene
Long March 5B	20	Wenchang	Cryogenic	Kerosene
Long March 7	20	Wenchang	Kerosene	Kerosene
Long March 7A	20	Wenchang	Kerosene	Kerosene
Long March 8	40	Wenchang	Kerosene	Kerosene
Minotaur-C	10	Vandenberg	Solid	Solid
Soyuz ST-A	10	Kourou	Kerosene	Kerosene

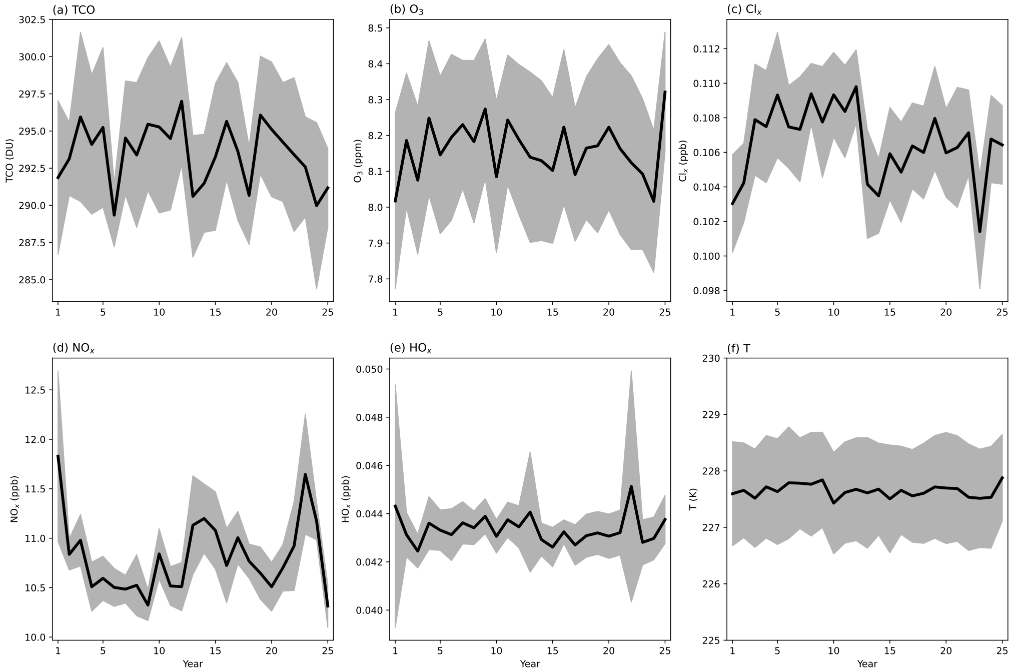

Figure 1: (a) Global-, annual-mean total column ozone (solid black line) plus/minus one standard deviation (grey shading) for each of the 25 years in the ALL simulation. (b)–(e): As for (a), but showing concentrations of ozone, reactive chlorine (Cl_x=Cl+ClO), nitrogen oxides (NO_x=NO+NO_2) and hydrogen oxides (HO_x=H+OH+HO_2) at 10 hPa. (f) As for (a) but showing temperature at 10 hPa.
